# Exogenous Application of Non-mature miRNA-Encoded miPEP164c Inhibits Proanthocyanidin Synthesis and Stimulates Anthocyanin Accumulation in Grape Berry Cells

**DOI:** 10.3389/fpls.2021.706679

**Published:** 2021-10-05

**Authors:** Mariana Vale, Jéssica Rodrigues, Hélder Badim, Hernâni Gerós, Artur Conde

**Affiliations:** ^1^Department of Biology, Centre of Molecular and Environmental Biology, University of Minho, Braga, Portugal; ^2^Centre for the Research and Technology of Agro-Environmental and Biological Sciences, University of Trás-os-Montes e Alto Douro, Vila Real, Portugal; ^3^Department of Biological Engineering, Centre of Biological Engineering, University of Minho, Braga, Portugal

**Keywords:** non-mature miRNA-encoded peptide, miRNA regulation, grape berry secondary metabolism, post-transcriptional regulation, anthocyanins

## Abstract

Secondary metabolic pathways in grape berries are tightly regulated by an array of molecular mechanisms, including microRNA-mediated post-transcriptional regulation. As recently discovered, before being processed into mature microRNAs (miRNAs), the primary transcripts of miRNAs (pri-miRNAs) can encode for small miRNA-encoded peptides (micropeptides – miPEPs) that ultimately lead to an accentuated downregulation of the respective miRNA-targeted genes. Although few studies about miPEPs are available, the discovery of miPEPs reveals a new layer of gene regulation at the post-transcriptional level that opens the possibility to regulate plant metabolism without resorting to gene manipulation. Here, we identified a miPEP encoded in non-mature *miR164c* putatively targeting grapevine transcription factor VvMYBPA1 (miPEP164c/miPEP-MYBPA1), a positive regulator of key genes in the proanthocyanidin (PA)-biosynthetic pathway, a pathway that competes directly for substrate with the anthocyanin-biosynthetic pathway. Thus, the objective of this work was to test the hypothesis that the exogenous application of miPEP164c (miPEP-MYBPA1) can modulate the secondary metabolism of grape berry cells by inhibiting PA biosynthetic pathway while simultaneously stimulating anthocyanin synthesis. The exogenous application of miPEP164c to suspension-cultured cells from grape berry (cv. Gamay) enhanced the transcription of its corresponding non-mature *miR164c*, with a maximum effect at 1 μM and after a period of 10 days, thus leading to a more pronounced post-transcriptional silencing of its target VvMYBPA1. This led to a significant inhibition of the PA pathway, mostly *via* inhibition of leucoanthocyanidin reductase (LAR) and anthocyanidin reductase (ANR) enzymatic activities and *VvLAR1* downregulation. In parallel, the anthocyanin-biosynthetic route was stimulated. Anthocyanin content was 31% higher in miPEP164c-treated cells, in agreement with the observed upregulation of *VvUFGT1* transcripts and UFGT enzyme activity levels.

## Introduction

Although grapevines are well adapted to semi-arid climate, the increasingly more frequent combined effect of drought, high air temperature and high evaporative demand has a negative impact in grapevine yield (Chaves et al., 2010) and, if severe, also in berry quality (Teixeira et al., 2013). Therefore, berry and wine quality depend strongly on the grapevine adaptability to drought, heat and light/UV intensity. This abiotic stressors particularly impact highly regulated molecular mechanisms underlying the synthesis of several quality-related compounds, such as anthocyanins, proanthocyanidins (PAs), flavanols, and flavonols (Downey et al., 2006; Teixeira et al., 2013).

Anthocyanin and PA (condensed tannins) biosynthetic pathways compete for two common precursors, leucoanthocyanidins and anthocyanidins (Li et al., 2016). Proanthocyanidins are composed of several monomers of (+)-catechin and (−)-epicatechin, both flavan-3-ols that originate in a branch deviation of the general flavonoid pathway. Catechin synthesis is catalyzed by leucoanthocyanidin reductase (LAR) that uses leucoanthocyanidins as substrate. However, leucoanthocyanidins can also be catalyzed by leucoanthocyanidin oxygenase (LDOX), continuing the flavonoid pathway and resulting in the formation of anthocyanidins, a substrate of both UDP-glucose flavonoid 3-O-glucosyltransferase (UFGT), in the synthesis of anthocyanins, and anthocyanidin reductase (ANR), in the synthesis of epicatechin, another building block of PAs (Gagné et al., 2009).

In grapevine, many transcription factors belonging to the R2R3-MYB family are involved in the regulation of flavonoid synthesis by inducing or silencing key biosynthetic genes along the flavonoid pathway (Deluc, 2006; Matus et al., 2009). The transcription factors VvMYB5a and VvMYB5b are already described as positive regulators of the flavonoid pathway, inducing an upregulation of late-stage berry-associated genes such as *VvCHI* (chalcone isomerase), *VvF3′5* (flavonoid 3′,5′-hydroxylase), *VvDFR* (dihydroflavonol 4-reductase), *VvLDOX*, *VvANR*, and *VvLAR1* leading to the synthesis of flavonols, anthocyanidins, and PAs (Cavallini et al., 2014; Pérez-Díaz et al., 2016). *VvMYBPA1*, expressed during flowering and early berry development, is a positive regulator of PA synthesis, by upregulating *VvLDOX*, *VvANR*, and *VvLAR1* genes (Bogs et al., 2007; Cavallini et al., 2015), thus limiting the progress of the anthocyanin-biosynthetic route.

Regulation of the flavonoid pathway can also be coordinated at the post-transcriptional level by several microRNAs (miRNAs; Xie et al., 2010) that negatively regulate the expression of their target genes, either by promoting degradation of such target messenger RNAs (mRNAs) or by leading to inhibition of targeted mRNA translation (Pantaleo et al., 2010). MicroRNAs are initially transcribed as much larger primary transcripts (pri-miRNAs) and only become mature miRNA after several maturation processes (Xie et al., 2010). Like any other protein-coding gene, miRNAs are transcribed by RNA polymerase II originating the primary transcript of miRNA (pri-miRNA) that consists of a few hundred bases, a 5′cap and 3′ poly-A tail and the characteristic stem-loop structure where the miRNA sequence is inserted, and which is recognized by members of the Dicer-like1 family enzymes. This enzyme cleaves the 5′cap and 3′ poly-A tail of the primary transcript, transforming it in a precursor miRNA (pre-miRNA). DCL1 also carries out the subsequent cleavage of pre-miRNA to release the miRNA:miRNA^∗^ duplex which is then translocated to the nucleus by HASTY transporter where the correct miRNA strand is incorporated in a ribonuclear particle to form the RISC complex, the machinery that mediates miRNA-mediated gene silencing (Budak and Akpinar, 2015).

In a groundbreaking finding, it was discovered that, before being processed into mature miRNAs, some pri-miRNAs contain small open reading frames (ORF) that could encode for small regulatory peptides called miRNA-encoded peptides (miPEPs; Lauressergues et al., 2015). The mechanism of action of miPEPs is by enhancing the transcription and accumulation of the corresponding pri-miRNA, in a sort of positive feedback loop, that subsequently results in accentuated downregulation of the respective miRNA-targeted genes (Couzigou et al., 2015). For instance, the overexpression of miPEP171b in *Medicago truncatula* led to the increased accumulation of endogenous miR171b (involved in the formation of lateral roots), which resulted in significant changes in root development (Couzigou et al., 2016). Moreover, in soybean (*Glycine max*), it was demonstrated for the first time that the exogenous application of well-chosen, synthetic miPEP172c had a positive impact in nodule formation, by inducing the overexpression of *pri-miR172c*, whose correspondent miR172c accumulation results in an increase in nodule formation and consequent improvement of nitrogen fixation (Couzigou et al., 2016).

More recently, a miPEP in *Arabidopsis*, miPEP858, was reported by screening the 1,000 bp region upstream of pre-miR858 for small ORFs (Sharma et al., 2020) miPEP858 was able to modulate the expression of targets gene involved in plant growth and development and also on the phenylpropanoid pathway, by inducing the expression of *pri-miR858*.

In a very recent finding, using a fluorescein-labeled peptide, it was demonstrated that exogenously applied miPEP-156a could effectively penetrate plant seedlings through the root system and disperse systemically to the leaves of young seedlings of *Brassica rapa* (Erokhina et al., 2021). The application of this miPEP exerted a moderate morphological effect consisting of a growth acceleration of the main root of the seedling, that was parallel to an increase in *pri-miR156a* expression. These morphological and molecular level effects were apparently related with the ability of the miPEP to rapidly transfer into the cell nuclei and bind to nuclear chromatin.

Screening for small ORFs, either in the precursor sequence of miRNA or in the region upstream of such precursor, is the mainstream method for finding putative miPEPs when a miRNA is available, or a targeted gene is in mind. Yet, alternative molecular approaches for screening for miPEPs have also been successful. These include homology based computational analysis using expressed sequence tags (ESTs) of a certain species genome by blasting it against miRNA sequences already described, to find homologous of miRNAs and then repeat the same methodologies of miRNA target prediction and screening for small ORFs in the pre-miRNA sequence (Ram et al., 2019).

Recently, a miPEP in grapevine was reported, miPEP171d1, originating from a non-mature miRNA conserved within different plant species and associated with root organ development. When exogenously applied, miPEP171d1 was able to promote adventitious root formation thus enabling to overcome challenges in clonal propagation (Chen et al., 2020).

Although few studies about miPEPs are available, the discovery of miPEPs reveals a new layer of gene regulation at the post-transcriptional level that opens the possibility to regulate plant metabolism without resorting to gene manipulation.

Taking these groundbreaking discoveries as basis, the objective of this work was to test the hypothesis that the exogenous application of a newly identified putative grapevine miPEP by our group (miPEP164c – miPEP-MYBPA1) can modulate the secondary metabolism of grape berry cells by inhibiting PA biosynthetic pathway while simultaneously stimulating anthocyanin synthesis. The micropeptide miPEP164c is putatively targeting MYBPA1, as predicted *in silico*, a gene encoding for a transcription factor that acts as a positive regulator of PA synthesis by activating the expression of *VvLAR* and *VvANR*, the genes encoding for the enzymes responsible for catechin and epicatechin synthesis (Bogs et al., 2007). For that, a wide array of molecular biology and classic biochemistry approaches were combined to better assess the impact of miPEP164c exogenous treatments on the transcription of key genes involved in secondary metabolic pathways, on the biochemical activity of the corresponding key enzymes, and on the final concentration of secondary metabolites such as anthocyanins and PAs.

## Materials and Methods

### *In silico* Analyses

A series of *in silico* analyses to identify potential miPEPs in grapevine by combining several bioinformatic tools and databases such as the bioinformatic tool psRNATarget Finder (Dai et al., 2018), a plant small regulatory RNA target predictor, with the aid of GenBank, was used to retrieve information on which, how and where (in the target RNA) miRNAs putatively regulated key genes directly or indirectly involved in the flavonoid biosynthetic pathway. We identified *miR164c* as a putative negative regulator of transcription factor VvMYBPA1, involved in regulation of the PA pathway and proceeded to screen it in miRbase (microRNA database; Kozomara and Griffiths-Jones, 2014) for their stem-loop sequence or pri-miRNA sequence, the non-mature sequence of the regulatory miRNA possibly harboring small ORFs corresponding to regulatory miPEPs. Finally, the obtained stem-loop sequence was then ran in a bioinformatic ORF finder tool that recognizes in the introduced sequence all possible ORFs that could translate into a small peptide, by defining several parameters based on the few miPEPs so far identified in the literature (Lauressergues et al., 2015). This several step analysis led to the identification of one ORF with 48 bp, encoding a putative miPEP of 16 amino acids, that was designated as miPEP164c.

### Solubilization of miRNA-Encoded Peptides

Following *in silico* identification, miPEP164c and a scrambled miPEP were ordered from Smart Bioscience as 1 mg aliquot. The scrambled miPEP aminoacidic sequence is the same as miPEP164c aminoacidic composition but in a random/scrambled order: MGTISKETCSQTNQCT. Solubilization of the micropeptides was conducted as recommended by Smart-Bioscience Peptide Solubility Guidelines.^1^ Both miPEPs were solubilized in 200 μL of acetic acid (10%) and 800 μL of DMSO to a final concentration of 1 mg/mL. A solution of 200 μL of acetic acid (10%) and 800 μL of DMSO was used as control in the exogenous treatments.

### Biological Material

Grape berry cell suspensions of the cultivar Gamay Freaux cv. were maintained in Gamborg B5 medium in 250 mL flasks at 25°C with constant agitation on a rotator shaker at 100 rpm and under 16 h/8 h photoperiod. The culture medium composition was as follows: 3 g/L Gamborg B5 salt mixture and vitamins; 30 g/L sucrose (3% m/v); 250 mg/L casein enzymatic hydrolysate; 0.1 mg/L α-naphthaleneacetic acid (NAA); 0.2 mg/L Kinetin, and a final pH of 5.7. The suspension-cultured cells were allowed to grow for 10-day, until the exponential phase, when they were subcultured by transferring 10 mL of cells to 30 mL of fresh medium.

### Exogenous Addition of Micropeptides to Gamay Grape Cells

Immediately after sub-cultivation, concentrations ranging from 0.1 to 2 μM of miPEP164c were added to the cell cultures, in a volume that represented no more than 0.15% (v/v) of the total volume of the cell suspension. All cell suspensions, including control cells (treated with the same volume of control solution) and cells treated with the scrambled miPEP (a control miPEP with the same aminoacidic composition as miPEP164c but with a scrambled amino acid sequence) were cultivated for 10-day with constant agitation and a 16 h/8 h photoperiod. A time-course analysis of 1 μM of miPEP164c with several time-points (1 h, 24 h, 3-, 5- and 10-day) was also conducted. Also, upon miPEP164c addition (*t* = 0 h), samples were taken for anthocyanin and PA base level quantifications. Cells were collected when necessary, filtered and immediately frozen with liquid nitrogen and stored at −80°C. A part of cells of each experimental condition was lyophilized.

### Quantification of Anthocyanins

Anthocyanins were extracted from 100 mg of grape berry cells from each experimental condition. After adding 1 mL of 90% methanol and 10% deionized H_2_O, the suspensions were vigorously shaken for 30 min, following centrifugation at 18000 × *g* for 20 min. The supernatants were collected and 200 μL of each supernatant was mixed with 1.8 mL of a solution of 25 mM KCl (pH = 1.0) and absorbance was measured at 520 and 700 nm. Total anthocyanin quantification was calculated in relation to cyanidin-3-glucoside (C-3-G) equivalents, as follows in the equation, and subsequently presented per dry weight (DW):


$$\begin{array}{c} \left[\mathrm{Total}\,\mathrm{anthocyanins}\right]\,\left(\mathrm{mg}/L\right)=\\ \frac{\left({\text{A}}_{520}-{\text{A}}_{700}\right)\times \mathrm{MW}\times \mathrm{DF}\times 1000}{\varepsilon \times 1} \end{array}$$

where MW is the molecular weight of C-3-G (449.2 g mol^–1^), DF is the dilution factor and ε is the molar extinction coefficient of C-3-G (26900 M^–1^ cm^–1^).

### Quantification of Proanthocyanidins

Proanthocyanidin content was determined using an adapted colorimetric vanillin-HCl assay described by Broadhurst and Jones (1978). To extract PAs, 1 mL of 100% methanol was added to 5 mg of lyophilized grape berry cells and vigorously shaken for 30 min followed by centrifugation at 18000 × *g* for 15 min. Supernatants were collected and diluted in a 1:1 ratio with methanol to final volume of 500 μL. The methanolic extracts were added to clean assay tubes wrapped with aluminum foil. Then, 3 mL of a solution of 4% (m/v) vanillic acid freshly prepared in methanol was added and mixed very gently. Finally, 1.5 mL of concentrated hydrochloric acid was added to each reaction tube and mixed very gently. The reactions were allowed to stand for 6 min and the absorbance of the samples was measured spectrophotometrically at 500 nm. To discard absorbance interference caused by anthocyanin presence in the samples, control reactions for each condition were prepared with 3 mL of methanol instead of vanillic acid and the absorbance measured at 500 nm was discounted from the absorbance of reaction mixtures with vanillic acid. An epigallocatechin gallate (EGCG) standard curve, with concentrations ranging from 10 to 200 μg employing the same method was always prepared for each quantification of PA content.

### Protein Extraction

Protein extraction was conducted as described in Conde et al. (2016). Lyophilized grape berry cells were mixed with extraction buffer in approximately 1:1 (v/v) powder: buffer ratio. Protein extraction buffer contained 50 mM Tris–HCl, pH 8.9, 5 mM MgCl_2_, 1 mM EDTA, 1 mM PMSF, 5 mM DTT, and 0.1% (v/v) Triton X-100. Homogenates were thoroughly vortexed and centrifuged at 18000 × *g* for 15 min at 4°C. Supernatants were maintained on ice and used for all enzymatic assays. Total protein concentrations of the extracts were determined by the method of Bradford (1976) using bovine serum albumin as a standard.

### Enzymatic Activity Assays

The biochemical activity of UFGT was determined as described by Lister et al. (1996) adapted by Conde et al. (2016). The assay mixture contained 385 μL of 100 mM Tris–HCl reaction buffer (pH 8), 100 μL of enzyme extract, 10 μL of 50 mM UDP-glucose and the reaction was initiated with 5 μL of 100 mM quercetin as substrate for the enzyme activity (saturating concentration) to a final reaction volume of 500 μL. Each mixture was incubated for 30 min in the dark with gentle shaking. After incubation, dilutions were prepared with 100 μL of each assay mixture and 900 μL of Tris–HCl reaction buffer and absorbance was read at 350 nm immediately after (*t* = 0) and 30 min later (*t* = 30) to follow the production of quercetin 3-glucoside (ε = 21877 M^–1^ cm^–1^).

Leucoanthocyanidin reductase enzymatic activity was measured by spectrophotometrically monitoring the conversion of dihydroquercetin to (+)-catechin following the method of Gagné et al. (2009) with some adaptions. The assay mixture contained 1.7 mL of Tris–HCl buffer (0.1 M, pH 7.5), 300 μL of protein extract, 2 μL of NADPH (100 mM) and the reaction was initiated by adding 1 μL of dihydroquercetin (10 mg mL^–1^ in DMSO). The production of (+)-catechin (ε = 10233 M^–1^ cm^–1^) was followed at 280 nm for 30 min.

The biochemical activity of ANR was determined as described by Zhang et al. (2012) with some adaptations. The assay mixture contained 1.5 mL of PBS buffer (0.1 M, pH 6.5), 60 μL of enzyme extract, 40 μL of ascorbic acid (20 mM), 50 μL of cyanidin chloride (2 mM) and the reaction was initiated by adding 75 μL of NADPH (20 mM) followed by a 1/10 dilution with PBS reaction buffer for proper absorbance measure. The enzyme activity was monitored by measuring the rate of NADPH (ε = 6.22 mM^–1^ cm^–1^) oxidation at 340 nm for 20 min, at 45°C.

### RNA Extraction and cDNA Synthesis

Total RNA extraction was performed according to Reid et al. (2006) in combination with purification steps from the GRS Total Plant RNA extraction kit. After treatment with DNase I (Qiagen), cDNA was synthesized from 1 μg of total RNA using Maxima first strand cDNA synthesis kit from Thermo Fisher Scientific, following the manufacturer’s instructions. RNA concentration and purity were determined using Nanodrop and its integrity assessed in a 1% agarose gel stained with SYBR Safe (InvitrogenTM, Life Technologies).

### Transcriptional Analyses by Real-Time qPCR

Quantitative real-time PCR was performed with QuantiTect SYBR Green PCR Kit (Qiagen) in a CFX96 Real-Time Detection System (Bio-Rad), using 1 μL of cDNA in a final reaction volume of 10 μL per well. Specific primer pairs used for each target gene are listed in Supplementary Table 1. Melting curve analysis was performed for specific gene amplification confirmation. As reference genes, *VvACT1* (actin) and *VvGAPDH* (glyceraldehyde-3-phosphate dehydrogenase) were selected, as these genes were proven to be very stable and ideal for qPCR normalization purposes in grapevine (Reid et al., 2006). For all experimental conditions tested, three biological replicates were used with internal triplicates. The expression values were normalized by the average of the expression of the reference genes as described by Pfaffl (2001) and analyzed using the software Bio-Rad CFX Manager (Bio-Rad).

### Statistical Analyses

The results were statistically analyzed by Student’s *t*-test using Prism vs. 6 (GraphPad Software, Inc.). For each condition, statistical differences between the mean values of miPEP-treated cells and control cells are marked with asterisks. For every experimental approach, three biological replicates (constituted by three independent treatments and respective experiments) were used.

## Results

### Identification and *in silico* Analysis of the Candidate Grapevine Micropeptide miPEP164c

An *in silico* analysis for micropeptide screening led to the selection of miPEP164c, a candidate miPEP with putative regulatory function in grape berry flavonoid biosynthesis metabolic pathway, particularly in the branch of PA synthesis. miR164c was predicted *in silico* to post-transcriptionally inhibit grapevine transcription factor *VvMYBPA1*, involved in the activation of flavonoid synthesis, specifically of PA synthesis (*via* LAR1, LAR2, and ANR activation). Relevant information obtained by the *in silico* analysis regarding the miPEP selected for this study, including its aminoacidic sequence, attributed name and respective mature miRNA name and miRbase accession number, as well as that of its precursor miRNA (pre-miRNA), is detailed in Table 1.

**TABLE 1:** Detailed information about the micropeptide identified by an *in silico* analysis and selected for this study and its corresponding mature miRNA and mode of action.

miPEP	Amino acid sequence	miRNA	Stem-loop sequence	Mode of action	Predicted target
miPEP164c (miPEP-MYBPA1)	MEKQGTCITSSCTTNQ	miR164c (MIMAT0005660)*	MI006505*	Inhibition in translation	*VvMYBPA1*

*Acession code for miRBase (Kozomara and Griffiths-Jones, 2014).

### Effect of miPEP164c Exogenous Application on the Abundance of Pre-miR164c and Its Putative Target Transcription Factor VvMYBPA1

**FIGURE 1:**
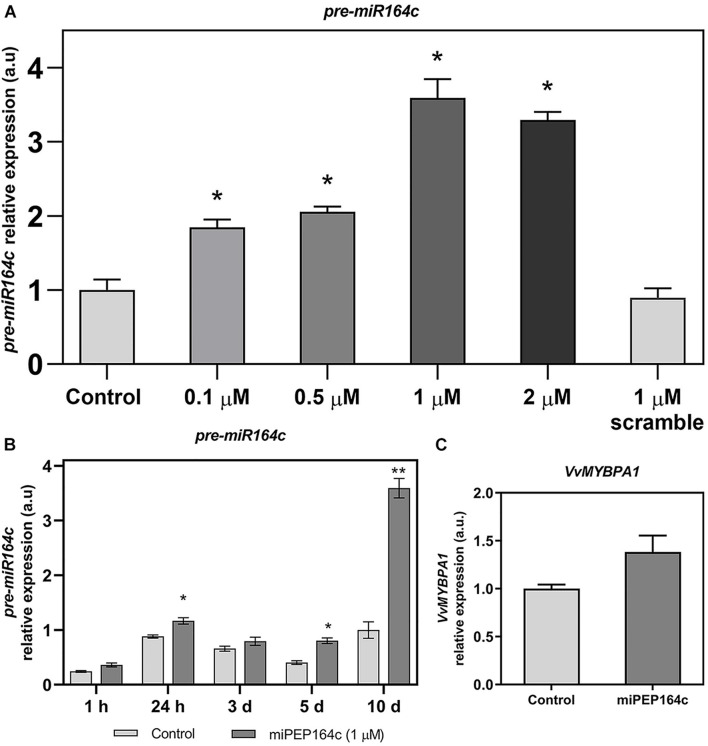
Steady-state transcript levels of *pre-miR164c* in suspension-cultured grape berry cells (cv. Gamay) 10-day after elicitation with various concentrations of miPEP164c **(A)** in suspension-cultured grape berry cells (cv. Gamay) eliciated with 1 μM of miPEP164c throughout a period of 10-day **(B)** and the steady-state transcript levels of *VvMYBPA1* **(C)** in suspension-cultured grape berry cells (cv. Gamay) 10 days after elicitation with 1 μM miPEP164c. Gene expression analysis, by real-time qPCR was normalized with the expression of reference gene *VvACT1* and *VvGAPDH*. Values are the mean ± SEM. Asterisks indicate statistical significance in relation to the control (Student’s *t*-test; *P < 0.05; **P < 0.001).

To confirm if miPEP164c exogenous application is indeed activating the accumulation of its *in silico* predicted miRNA (*miR164c*), gene expression analysis by real-time qPCR of the non-mature pre-miR164c was performed on cells treated with different concentrations of miPEP164c, ranging from 0.1 to 2 μM. As shown in Figure 1A, 10 days after treatment, the transcript levels of *pre-miR164c* were increasingly upregulated along miPEP concentration reaching a maximum effect at 1 μM (3.5-fold increase) that was approximately maintained at 2 μM of miPEP164c (3.2-fold increase). The exogenous application of a scrambled miPEP at 1 μM did not result in any changes in transcript levels of *pre-miR164c* after 10 days of treatment.

**FIGURE 2:**
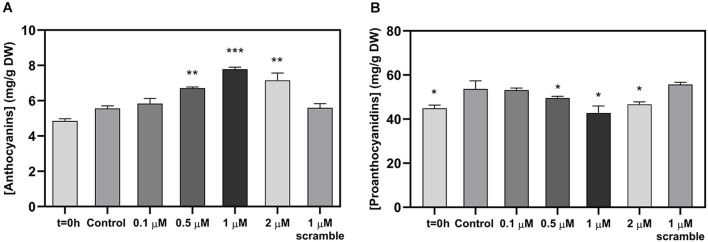
Effect of the exogenous application of different concentrations of miPEP164c on total anthocyanin content **(A)** and on total proanthocyanidin (PA) content **(B)** in suspension-cultured grape berry cells (cv. Gamay) 10-day after elicitation with miPEP164c. Anthocyanin concentration is represented as mg of cyanidin-3-glucoside (C-3-G) equivalents per g of fresh weight (FW). Asterisks indicate statistical significance in relation to the control (Student’s *t*-test; *P < 0.05; **P < 0.01; ***P < 0.001).

A time-course analysis of the transcript levels of *pre-miR164c* in cells treated with 1 μM of miPEP164c through a period of 10 days (1 h, 24 h, 3-, 5- and 10-day) confirmed, as shown in Figure 2A, that the expression of *pre-miR164c* was slightly stimulated at 24 h and 5-day of miPEP164c treatment, but had the highest increase (3.5 fold) after 10 days of treatment (Figure 1B).

No statistically significant change in the transcript levels of *VvMYBPA1* was observed after 10 days of miPEP164c treatment (Figure 1C).

### Effect of miPEP164c Exogenous Application on Grape Berry Key Secondary Metabolites

Spectrophotometric quantifications of grape berry cells secondary metabolites revealed a significant increase in anthocyanin content at 0.5, 1, and 2 μM of miPEP164c after 10 days of treatment with the highest concentration of anthocyanins quantified in cells treated with 1 μM miPEP164c, with 7.4 mg of total anthocyanins per g of dry weight compared to only 5.6 mg per g of dry weight in control cells, which represented a 31% increase (Figure 2A).

In parallel, PAs concentration decreased significantly at those same concentrations of 0.5, 1, and 2 μM of miPEP164c with a more representative decrease (26%) from 54 mg of PAs per g of dry weight in control cells to 42.7 mg of PAs per g of dry weight after 10 days of treatment with 1 μM miPEP164c (Figure 2B). The exogenous application of 1 μM of the scrambled miPEP did not change the concentrations of both anthocyanins and PAs.

### Transcriptional and Biochemical Changes Induced by miPEP164c on the Proanthocyanidin-Synthesizing Branch

**FIGURE 3:**
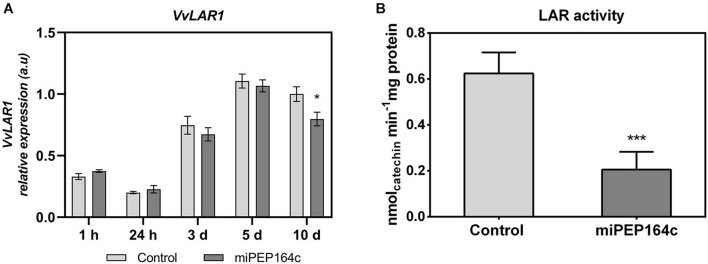
Steady-state transcript levels of *VvLAR1* in suspension-cultured grape berry cells (cv. Gamay) 10-day after elicitation with various concentrations of miPEP164c **(A)** and the effect on the specific activity of leucoanthocyanidin reductase (LAR) **(B)** in suspension-cultured grape berry cells (cv. Gamay) after 10 days of elicitation with 1 μM miPEP164c. Gene expression analysis, by real-time qPCR was normalized with the expression of reference gene *VvACT1* and *VvGAPDH*. Values are the mean ± SEM. Asterisks indicate statistical significance in relation to the control (Student’s *t*-test; *P < 0.05). LAR biochemical activity represented as the *V*_*max*_ in grape berry cells under miPEP164c treatment. Values are the mean ± SEM. Asterisks indicates statistical significance (Student’s *t*-test; ***P < 0.001).

**FIGURE 4:**
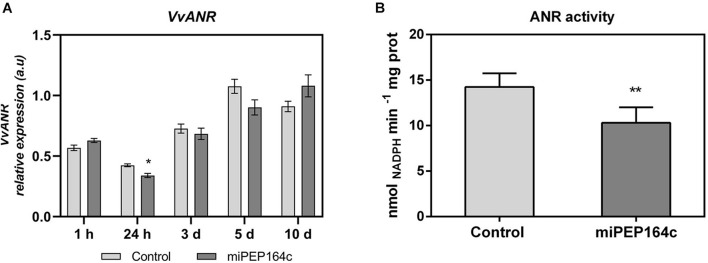
Steady-state transcript levels of *VvANR* in suspension-cultured grape berry cells (cv. Gamay) 10-day after elicitation with various concentrations of miPEP164c **(A)** and the effect on the specific activity of anthocyanidin reductase (ANR) **(B)** in suspension-cultured grape berry cells (cv. Gamay) after 10 days of elicitation with 1 μM miPEP164c. Gene expression analysis, by real-time qPCR was normalized with the expression of reference gene *VvACT1* and *VvGAPDH*. Values are the mean ± SEM. Asterisks indicate statistical significance in relation to the control (Student’s *t*-test; *P < 0.05). ANR biochemical activity represented as the *V*_*max*_ in grape berry cells under miPEP164c treatment. Values are the mean ± SEM. Asterisks indicates statistical significance (Student’s *t*-test; **P < 0.01).

A time-course analysis by real-time qPCR on *VvLAR1* expression in cells treated with 1 μM of miPEP164c showed a significant decrease of 20% after 10-day, while no statistically significant changes were observed on earlier time-points (Figure 3A). In agreement with the observed decrease in the *VvLAR1* transcripts, LAR enzyme activity was 3-fold reduced in Gamay cells elicited with 1 μM miPEP164c, decreasing from a *V*_*max*_ of 0.63 nmol min^–1^ mg protein^–1^ in control cells to a *V*_*max*_ of 0.20 nmol min^–1^ mg protein^–1^ in miPEP-treated cells (Figure 3B). Regarding *VvANR* transcript levels, although there was a slight decrease at 24 h of miPEP exposure (20%) when compared to control cells, no changes were observed at other time-points (Figure 4A). However, a significant decrease of 27% was observed in the enzyme activity of ANR in cells treated with 1 μM miPEP164c for 10-day, from a *V*_*max*_ of 14 nmol min^–1^ mg protein^–1^ in control cells to 10.31 nmol min^–1^ mg protein^–1^ in miPEP-treated cells (Figure 4B).

### Transcriptional and Biochemical Changes Induced by miPEP164c on the Anthocyanin-Synthesizing Branch

**FIGURE 5:**
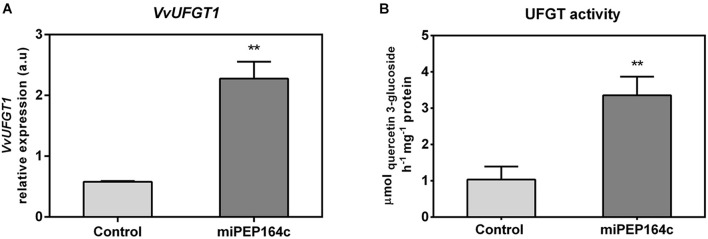
Steady-state transcript levels of *VvUFGT1* **(A)** in suspension-cultured grape berry cells (cv. Gamay) 10-day after elicitation with 1 μM miPEP164c and the effect on the specific activity of UDP-glucose flavonoid 3-O-glucosyltransferase (UFGT). **(B)** Gene expression analysis, by real-time qPCR was normalized with the expression of reference gene *VvACT1* and *VvGAPDH*. Values are the mean ± SEM. Asterisks indicate statistical significance (Student’s *t*-test; **P < 0.01). UFGT biochemical activity, represented as the *V*_*max*_ in grape berry cells under miPEP164c treatment. Values are the mean ± SEM. Asterisks indicates statistical significance (Student’s *t*-test; **P < 0.01).

The expression of *VvUFGT1* was strongly stimulated by miPEP164c application, reflected by a 4-fold increase in the expression levels in grape berry cells under this treatment (Figure 5A). The biochemical activity of UFGT was 3.2-fold higher in miPEP164c treated cells, in agreement with the transcriptional analysis, reaching a *V*_*max*_ of 3.4 μmol h^–1^ mg protein^–1^ (Figure 5B), which corroborates with a significant increase in the total concentration of anthocyanins observed previously (Figure 2A).

**FIGURE 6:**
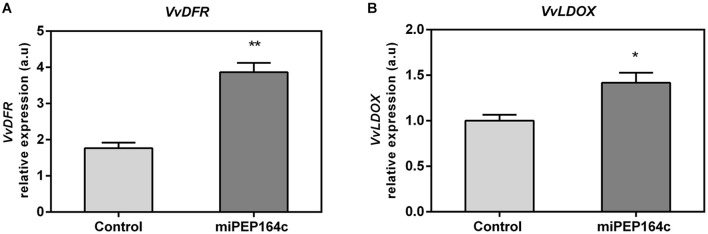
Steady-state transcript levels of *VvDFR* **(A)** and *VvLDOX* **(B)** in suspension-cultured grape berry cells (cv. Gamay) 10-day after elicitation with 1 μM miPEP164c. Gene expression analysis, by real-time qPCR was normalized with the expression of reference gene *VvACT1* and *VvGAPDH*. Values are the mean ± SEM. Asterisks indicate statistical significance (Student’s *t*-test; *P < 0.05; **P < 0.01).

The expression levels of *VvDFR* were also significantly stimulated, with a 2-fold increase in grape berry cells 10 days after miPEP164c treatment (Figure 6A). *VvLDOX* expression was also significantly stimulated by 42% when compared to control cells (Figure 6B).

**FIGURE 7:**
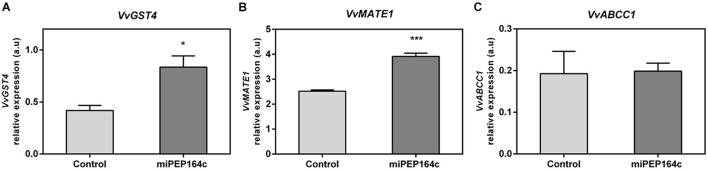
Steady-state transcript levels of *VvGST4* **(A)**, *VvMATE1* **(B)** and *VvABCC1* **(C)** in suspension-cultured grape berry cells (cv. Gamay) 10-day after elicitation with 1 μM miPEP164c. Gene expression analysis, by real-time qPCR was normalized with the expression of reference gene *VvACT1* and *VvGAPDH*. Values are the mean ± SEM. Asterisks indicate statistical significance (Student’s *t*-test; *P < 0.05; ***P < 0.001).

Transcriptional analysis showed that the expression of *VvGST4* under miPEP164c treatment also increased 2-fold (Figure 7A). Similarly, the transcript levels of *VvMATE1* increased by 55% (Figure 7B), while the expression of *VvABCC1* seemed not to be affected by treatment with this micropeptide (Figure 7C). These genes encode transporters that accumulate anthocyanins in the vacuole.

## Discussion

Grape berry secondary metabolism generates, by a cascade of reactions scattered through different branches of the phenylpropanoid pathway, a wide range of bioactive compounds with key roles in plant defense responses and with several health-related benefits to humans, making them metabolites of interest for many industries (Teixeira et al., 2013). Therefore, the search for new strategies to modulate these complex pathways in the hopes of either minimizing the effects of several stress factors in the composition and quality of grape secondary metabolites or to increase the synthesis and accumulation of bioactive metabolites of interest such as antioxidant compounds like anthocyanins, is a research line of great importance, not only to the viticulture industry but also in several industries of health-promoting products (Zhang et al., 2015). In the present study, we sought to validate a new and promising strategy to modulate the secondary metabolism of Gamay grape berry cells by testing a synthetic miPEP, putatively enhancing the transcription and accumulation of *miR164c* and ultimately promoting a more pronounced silencing of its predicted target. Because this transcription factor is involved in the molecular activation of key genes in the PA pathway, ultimately, we wanted to evaluate if a miPEP-based treatment could regulate grape berry secondary metabolism, by activating miRNA-mediated post-transcription silencing mechanisms of specific targets. Results obtained were very promising as this treatment could represent an innovative and easy-to-apply strategy to modulate the synthesis of more quality-related compounds, resulting in crops with added-value characteristics, without the need for more drastic, time consuming and more expensive strategies, as genetic transformation of crops.

### Elicitation of Gamay Cells With miPEP164c Induces Accumulation of miR164c and Consequent miR164c-Mediated Inhibition of Proanthocyanidin Biosynthetic Pathway

Overall, results confirmed that the exogenous application of miPEP164c is indeed enhancing the accumulation of *miR164c* which putatively resulted in a more pronounced post-transcriptional silencing of transcription factor VvMYBPA1 and consequently of MYBPA1-activated genes, here observed by a downregulation of *VvLAR1* expression and of LAR and ANR specific activity resulting in a significant decrease of 26% of total PA content in cells exposed to exogenously applied miPEP164c for 10-day. According to our results, the regulatory effect of miPEP164c, both at anthocyanin/PA concentrations and transcriptional levels, was most significant at a concentration of 1 μM and after a period of 10-day. This suggests that the regulatory effect of miPEP164c is cumulative along a period of exposure to the peptide, at least until 10-day, but not evident in a period of hours after the peptide treatment. It is possible that this cumulative effect after a longer period of exposure is due to the several molecular steps in the mechanistic underlying miPEP regulation intracellularly and the fact that miPEP164c does not directly regulate ultimate targets as LAR and ANR within PA-synthetic pathway, which probably results in more time needed to observe its regulatory effect.

Indeed, gene expression analysis by real-time qPCR confirmed that miPEP164c increased the expression levels of the *pre-miR164c* in Gamay cells along its concentration until 1 μM in an overwhelmingly more evident mode after 10-day. Thus, a positive loop was established, in which a consequent increased translation into miPEP164c, ultimately results in higher levels of mature miR164c and accentuated negative regulation of the target gene *VvMYBPA1. In silico* analyses suggests that the mode of action of *miR164c* is through inhibition of translation of *VvMYBPA1*, not by cleavage of the target messenger RNA, due to a lack of 100% complementarity between the guide miRNA and the target mRNA (Waterhouse and Hellens, 2015). This is in agreement with our results showing that the treatment with miPEP164c did not induce any significant changes in the expression levels of *VvMYBPA1.*

Evidence for the involvement of post-transcriptional silencing of *VvMYBPA1* mediated by miPEP164c was obtained when the MYBPA1-activated enzymes *VvLAR* and *VvANR* were clearly down-regulated. Both *VvANR* and *VvLAR1*, are key genes leading to the synthesis of PAs (Gagné et al., 2009). However, the expression of *VvANR*, encoding for the enzyme that synthesizes epicatechins from anthocyanidins, was not affected after 10-day of treatment, possibly to compensate the decreased activity of VvLAR, in order to ensure a certain amount of monomers for PAs biosynthesis. Also, *VvANR* expression may be regulated by several other regulatory proteins, such as *VvMYC1*, a bHLH transcription factor that physically interacts with MYB-like transcription factors like MYBPA1 and MYB5a/b to coordinate the regulation of *VvANR*, and therefore silencing of one transcription regulator may be overcome by another regulatory mechanism (Heppel, 2010).

### Proanthocyanidin Synthesis Was Inhibited by miPEP164c While Anthocyanin Synthesis Was Simultaneously Increased

The observed significant increase in anthocyanin total content in Gamay cells mediated by the application of miPEP164c corroborates our hypothesis that a miPEP164c-mediated silencing of PA synthesis would divert the carbon flow to the anthocyanin branch, due to the constant competition of both pathways for the same substrates, as reported before (Liao et al., 2015). Gene expression analysis of *VvUFGT1*, that glycosylates anthocyanidins into anthocyanins, revealed a strong upregulation of its expression levels in response to the elicitation with the micropeptide which goes in agreement with the observed increase of the UFGT specific activity that also increased.

In *Vitis vinifera* two types of anthocyanin tonoplast transporters that accumulate anthocyanins in the vacuole were identified: primary transporters from the ATP-binding cassette (ABC) family, such as the *VvABCC1* who requires the presence of reduced glutathione (GSH) to properly transport anthocyanins, through the tonoplast, into the vacuole (Jiang et al., 2019); and tonoplast secondary transporters like *VvMATE1* (anthoMATE) of the multidrug and toxic extrusion family that use the H^+^ gradient to transport mostly acylated anthocyanins (Gomez et al., 2009). Also crucial for anthocyanin stabilization and transport are the glutathione S-transferases, as the paradigmatic case of grapevine’s *VvGST4*, to promote anthocyanin S-conjugation with reduced glutathione for anthocyanin-stabilization purposes (Conn et al., 2008). Several studies on the role of GSTs in anthocyanin accumulation have described GSTs as escort/carrier proteins, binding anthocyanins to form a GST-anthocyanin complex, protecting them from oxidation and guiding anthocyanins from the cytosolic surface of the ER to the vacuole for proper storage mediated by tonoplast transporters such as *VvMATE1* and *VvABCC1* (Zhao and Dixon, 2010; Jiang et al., 2019). Our results strongly supported that anthocyanin transport capacity to the vacuole, where they are stored in grape berry cells, was also stimulated by miPEP164c application as the expression of the anthocyanin tonoplast transporter *VvMATE1* and anthocyanin carrier protein *VvGST4*, was upregulated by this micropeptide. It is not understood how plants choose between ATP-hydrolysis-dependent or H+/Na+-gradient dependent mechanisms for transport of native metabolites or xenobiotics. However, it is believed that the conjugation ligands, such as glucose or glutathione, play a key role in the determination of which transport mechanism will be used (Zhao and Dixon, 2010). However, the expression of *VvABCC1* was not affected by miPEP164c contrarily to what would be expected considering the upregulation of *VvGST4* expression. This could be due to the presence of other regulatory proteins affecting the expression of *VvABCC1*, other phenolic substrates that also need to be transported by this mechanism, the majority of anthocyanins might not be in the glycosylated form, which is the preferred form of anthocyanins of this type of transporter, or simply because it is competing with the upregulated *VvMATE1* transporter for anthocyanins (Francisco et al., 2013).

## Conclusion

In this study, recurring to a combination of molecular and biochemical approaches, we revealed that miPEP164c exogenous application induced a strong up-regulation of genes involved in anthocyanin synthesis, transport, and accumulation in the vacuole. Additionally, miPEP164c provoked a downregulation of PA synthesis (a pathway that directly competes with the anthocyanin-biosynthetic pathway), due to a decrease in *VvLAR1* expression levels with a corresponding very significant decrease in LAR total biochemical activity, accompanied also by a downregulated ANR biochemical activity.

**FIGURE 8:**
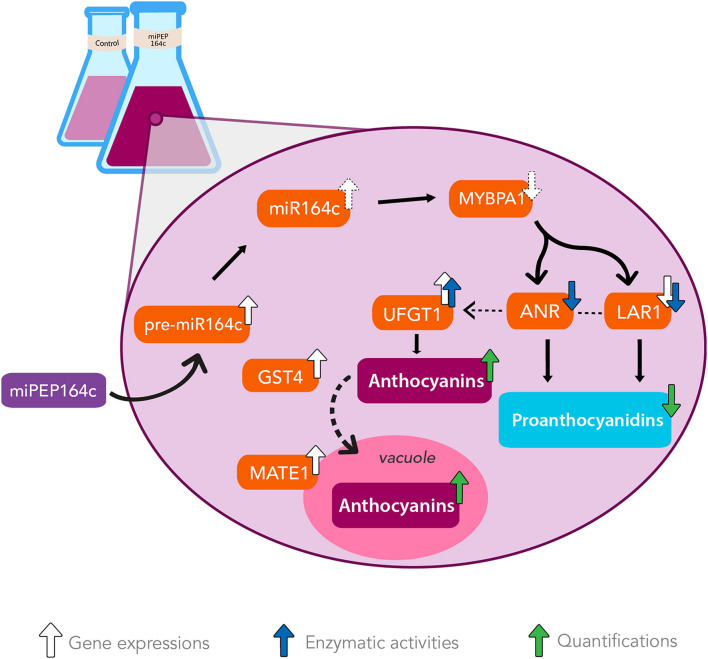
The exogenous application of miPEP164c increases anthocyanin synthesis and accumulation while decreasing PA synthesis by enhancing *miR164c*-mediated downregulation of PA-synthetic pathway in Gamay grape berry cell suspensions. Addition of miPEP164c provoked an increase in the transcription of pre-miR164c and consequently of its mature form, miR164c, which putatively led to a decrease in the translation of transcription factor MYBPA1. This inhibition in MYBPA1 translation resulted in a downregulation of *VvLAR1* expression and LAR total biochemical activity, as well as ANR total biochemical activity, decreasing the intracellular concentration of PAs. This downregulation of the PA pathway indirectly led to the stimulation of the anthocyanin synthesis by increasing *VvUFGT1* expression and UFGT total biochemical activity, as well as vacuolar accumulation, as shown by *VvGST4* and *VvMATE1* overexpression.

This upregulation of the anthocyanin biosynthetic route seems to be an indirect effect of miPEP164c putatively inhibiting transcription factor MYBPA1, a known positive regulator of PA synthesis. Thus, these metabolic alterations triggered by miPEP164c clearly resulted in higher concentration of anthocyanins and lower concentration of PAs, due to miR164c-mediated negative regulation of PA-related transcription factor *VvMYBPA1* and, consequently, *VvLAR1* and *VvANR*, ultimately leading to PA synthesis inhibition and anthocyanin synthesis stimulation as these pathways directly compete for substrate, in a mechanism illustrated in Figure 8.

## Data Availability Statement

The original contributions presented in the study are included in the article/Supplementary Material, further inquiries can be directed to the corresponding authors.

## Author Contributions

MV performed the experiments and wrote the manuscript. JR and HB performed the experiments. HG advised, wrote, and reviewed the manuscript. AC conceptualized the work, performed the experiments, and wrote and reviewed the manuscript. All authors contributed to the article and approved the submitted version.

## Conflict of Interest

The authors declare that the research was conducted in the absence of any commercial or financial relationships that could be construed as a potential conflict of interest.

## Publisher’s Note

All claims expressed in this article are solely those of the authors and do not necessarily represent those of their affiliated organizations, or those of the publisher, the editors and the reviewers. Any product that may be evaluated in this article, or claim that may be made by its manufacturer, is not guaranteed or endorsed by the publisher.

## Footnotes

1
https://www.smart-bioscience.com/support/solubility/
