# Supplementary material for: Exogenous Application of Non-mature miRNA-Encoded miPEP164c Inhibits Proanthocyanidin Synthesis and Stimulates Anthocyanin Accumulation in Grape Berry Cells
Source: Front Plant Sci. 2021 Oct 5;12:706679. doi: 10.3389/fpls.2021.706679 (PMC8523857; doi:10.3389/fpls.2021.706679)
Supplement: Supplementary file 1 [file Data_Sheet_1.PDF]

Supplementary Material

Supplementary table 1. Primers forward (F) and reverse (R) used for gene expression analysis by qPCR.

Gene	Acession number	Primers	Ref.
VvPAL1	GSVIVG01025703001	F: 5′-CCGAACCGAATCAAGGACTG-3′	Boubakri et al. (2013)
		R: 5′-GTTCCAGCCACTGAGACAAT-3′	
VvSTS1	GSVIVT0101059001	F: 5′-CGAAGCAACTAGGCATGTGT-3′	Boubakri et al. (2013)
		R: 5′-CTCCCCAATCCAATCCTTCA-3′	
VvCHS1	GSVIVT01032968001	F: 5′-GTCCCAGGGTTGATTTCCAA-3′	Boubakri et al. (2013)
		R: 5′-TCTCTTCCTTCAGACCCAGTT-3′	
VvCHS3	GSVIVT01000521001	F: 5’-GAAGTCGGCTGAGGAAGGGCTGAAGACC-3’	Bonghi et al. (2012)
		R: 5’-TCAACAGTGAGCCCTGGTCCGAAAC-3’	
VvFLS1	GSVIVT01008913001	F: 5’-CAGGGCTTGCAGGTTTTTAG-3’	Downey et al. (2003)
		R: 5’-GGGTCTTCTCCTTGTTCACG-3'	
VvDFR	GSVIVT01009743001	F: 5′-GGCTTTCTAGCGAGAGCGTA-3′	Bogs et al. (2006)
		R: 5′-ACTCTCATTTCCGGCACATT-3′	
VvLDOX	GSVIVT01032809001	F: 5’-ACCTTCATCCTCCACAACAT – 3’	Bogs et al. (2005)
		R: 5’-AGTAGAGCCTCCTGGGTCTT – 3’	
VvLAR1	GSVIVT01011958001	F: 5’-CAGGAGGCTATGGAGAAGATAC – 3’	Bogs et al. (2005)
		R: 5’-ACGCTTCTCTCTGTACATGTTG – 3’	
VvANR	GSVIVT01006396001	F: 5’-CAATACCAGTGTTCCTGAGC – 3’	Bogs et al. (2005)
		R: 5’-AAACTGAACCCCTCTTTCAC – 3’	
VvUFGT1	GSVIVT01024419001	F: 5’-TGCAGGGCCTAACTCACTCT-3’	Designed with the aid of QuantiPrime (Arvidsson et al. (2008))
		R: 5’-GCAGTCGCCTTAGGTAGCAC-3’	
VvGST4	GSVIVT01035256001	F: 5’-AAGGATCCATGGTGATGAAGGTGTATGGC-3'	Conn et al. (2008)
		R: 5’-AACTGCAGAAGCCAACCAACCAACAAAC-3’	
VvMATE1	GSVIVT01028885001	F: 5’-TGCTTTTGTGATTTTGTTAGAGG-3’	Gomez et al. (2009)
		R: 5’-CCCTTCCCCGATTGAGAGTA-3’	
VvABCC1	GSVIVT01028722001	F: 5’-CTCCACTGGTCCTCTGCTTC-3’	Designed with the aid of QuantiPrime (Arvidsson et al. (2008))
		R: 5’-AGCCTGCTTCGAAAGTACCA-3’	
VvMYBPA1	GSVIVT01027182001	F: 5’-AGATCAACTGGTTATGCTTGCT-3	Bogs et al. (2007)
		R: 5’-AACACAAATGTACATCGCACAC-3	
Vvpre-miR164c	NR_127751	F: 5’-TTGAGCAAGATGGAGAAGCA – 3’	Designed in this study
		R: 5’-ATTGGTTTGTGGTGCATGAG – 3’	
VvActin	GSVIVT01026580001	F: 5’-GTGCCTGCCATGTATGTTGCC-3’	Conde et al. (2015)
		R: 5’-GCAAGGTCAAGACGAAGGATA-3’	
VvGADPH	GSVIVT00009717001	F: 5’-CACGGTCAGTGGAAGCATCAT-3’	Conde et al. (2015)

		R: 5’-CCTTGTCAGTGAACACACCAG-3’	
